# Evaluating cellular communication sensing for lapse risk prediction during early recovery from alcohol use disorder: A longitudinal observational study

**DOI:** 10.1371/journal.pone.0355396

**Published:** 2026-08-21

**Authors:** Kendra Wyant, Jiachen Yu, John J. Curtin

**Affiliations:** Department of Psychology, University of Wisconsin-Madison, Madison, Wisconsin, United States of America; NYU Grossman School of Medicine: New York University School of Medicine, UNITED STATES OF AMERICA

## Abstract

Alcohol Use Disorder (AUD) is a chronic, relapsing condition, and identifying periods of elevated lapse risk remains a major challenge in supporting recovery. An automated recovery monitoring and support system using personal sensing and machine learning may help detect when individuals are at heightened risk. Cellular communication sensing may be a promising approach for passively capturing risk-relevant information about social interactions, particularly when these data are contextualized with participant-specific meaning. We evaluated a machine learning model predicting next-day alcohol lapse among individuals in early recovery from AUD using contextualized cellular communication data and baseline alcohol use, demographic, and psychiatric and personality characteristics. A total of 144 participants (49% male; mean age = 40; 87% non-Hispanic White) with a goal of abstinence provided cellular communication data and alcohol use reports via a 4x daily EMA for up to three months. Models were trained and evaluated using repeated k-fold cross-validation. The best-performing full model used an elastic net algorithm and retained 10 features (median posterior auro C = 0.67, 95% Bayesian credible interval (CI; [0.64, 0.71]). A comparison model including only baseline features demonstrated comparable performance (median auROC = 0.69, 95% CI [0.65, 0.72]). Cellular communication features on their own performed poorly, but still above chance performance (median auROC = 0.59, 95% CI [0.55, 0.62]). These findings demonstrate that cellular communication data capture some risk-relevant signal for alcohol lapse but do not provide incremental predictive value beyond baseline measures. Nevertheless, several communication features were retained in the full model with moderately sized coefficients, suggesting that aspects of social communication may still be clinically relevant for understanding lapse risk. However, limitations inherent to cellular communication sensing may outweigh its added utility in lapse prediction models.

## Introduction

Alcohol Use Disorder (AUD) is a chronic, relapsing disease [1–3]. Lapses, single episodes of alcohol use, are among the strongest predictors (and a necessary precursor) of relapse, a full return to harmful drinking [4,5]. While lapses can occur at any point in recovery, they are particularly risky during early recovery [6]. Protective coping mechanisms and socio-environmental resources that support recovery are dynamic and accumulate over time [7]. Consequently, early recovery represents a critical window of vulnerability during which a lapse is more likely to escalate into relapse.

Given the dynamic and context-dependent nature of lapse risk, there is a clear need for methods capable of identifying when and why an individual is at increased risk in real time. An automated recovery monitoring and support system powered by personal sensing and machine learning may assist with this inherently difficult task. Personal sensing of densely sampled data from individuals’ day-to-day lives can provide the inputs necessary for temporally dynamic lapse prediction [8]. Early machine learning models using ecological momentary assessment (EMA) data have achieved excellent accuracy in predicting future lapses back to alcohol use in treatment seeking populations [9–11].

Despite the high predictive success of EMA-based approaches, questions remain about the long-term feasibility of a self-report sensing method. EMA has been shown to be well tolerated among substance-using populations over relatively short periods [12,13]. However, it is unclear whether individuals would be willing or able to adhere to intensive EMA protocols (e.g., four prompts per day) indefinitely. Moreover, EMA items are selected using domain expertise from decades of research on the self-report factors associated with lapse. It is possible, however, that additional lapse precipitants remain undiscovered due to small subtle changes in one’s environment, social circle, or lifestyle that cannot be easily detected via self-report.

Cellular communication sensing may be a promising alternative or complement to EMA. Whereas EMA is limited to, at most, several assessments per day, communication sensing is mostly passive and can be monitored moment-by-moment. Cellular communication patterns capture clear, risk-relevant constructs. Late-night phone calls could indicate an emergency, “drunk dialing,” or interpersonal conflict. A decrease in the number of contacts an individual communicates with could reflect a shrinking social circle, isolation, or disengagement. Furthermore, cellular communication sensing enables data-driven feature engineering, whereby features are systematically derived from raw communication logs and retained based on their predictive utility rather than a priori theoretical assumptions.

These data may become even more powerful when communication patterns are contextualized with participant-specific meaning. Knowing a participant’s relationship to their contacts, whether they have previously drunk alcohol with a given contact, or whether that contact supports their recovery goals could fundamentally alter interpretation. In the examples above, contextualized communication data might reveal that the late-night calls are made to a sponsor, or that a shrinking social circle reflects reduced contact with individuals unsupportive of their recovery. In this way, the same communication patterns may reflect protective processes rather than increased lapse risk.

This focus on contextualized communication is especially important because lapse occurs within one’s social context. A substantial body of literature demonstrates that social support and social network influences play a central role in drinking behavior and recovery outcomes [14–16]. Positive social relationships have consistently been associated with better alcohol treatment outcomes [17–20]. For example, the supportive and pro‑abstinent social environment of self‑help groups, like Alcoholics Anonymous, is thought to play a major role in their efficacy [18,21–23]. Conversely, maintaining relationships with heavy drinkers or individuals unsupportive of recovery is associated with poorer outcomes [24–26]. The buffering hypothesis further suggests that positive social relationships mitigate stress by providing resources that promote adaptive responses [27,28], a mechanism that likely extends to lapse risk given stress’s complex relationship with substance use [29].

Importantly, these influences unfold over time through everyday social interactions. Because many social interactions occur over the phone, cellular communication logs provide a natural window into patterns of social engagement. Social relationships are inherently dynamic [30–33], and time-stamped communication data allow for the tracking of how these interactions fluctuate over time.

In this study, we evaluated whether contextualized cellular communication features contain clinically meaningful signals for predicting next-day alcohol lapse risk among individuals in early recovery from AUD. Using a machine learning approach, we examined the predictive utility of these features and identified the most important communication-based features, with the goal of uncovering novel, clinically relevant predictors of lapse risk.

## Methods

### Transparency and openness

We adhere to research transparency principles that are crucial for robust and replicable science. First, we reported how we determined the sample size, all data exclusions, all manipulations, and all study measures. We provide a transparency checklist [34] in the supplement. Second, our features, labels, questionnaires, code, and other study materials are publicly available on our Open Science Framework (OSF) page (https://osf.io/wgpz9/).

### Participants and procedure

We recruited 192 adults in early recovery from AUD in Madison, Wisconsin, USA through print and digital advertisements and partnerships with treatment centers from February 15, 2017 through September 19, 2019. This sample size was determined based on traditional power analysis methods for logistic regression [35] because comparable approaches for machine learning models have not yet been validated. Eligibility criteria required that participants were age 18 or older, able to read and write in English, had moderate to severe AUD (≥4 self-reported DSM-5 symptoms), had a goal of abstinence from alcohol at the time of the screening visit, had been abstinent for 1–8 weeks, were willing to use a single smartphone, and were not exhibiting severe psychosis or paranoia (defined as scores >2.2 or 2.8, respectively, on the psychosis or paranoia scales of the Symptom Checklist–90 [36]).

Participants completed up to 5 study visits over approximately 3 months: a screening visit, intake visit, and 3 monthly follow-up visits. At screening we determined eligibility and collected demographic information (age, sex at birth, race, ethnicity, education, marital status, and income) and clinical characteristics (DSM-5 AUD symptom count, personality [37], and psychiatric symptoms [36,38]). At the intake visit, approximately two weeks after screening, we collected additional self-report data on abstinence self-efficacy [39], craving [40], and recent recovery efforts and goals.

At each monthly follow-up, we downloaded backups of participants’ cellular communication metadata directly from their smartphones. Metadata included the phone number of the other party, the date and time of the communication, the origin of call or message (i.e., incoming or outgoing), whether the call was answered (voice calls only), and the duration of the call (voice calls only). During each follow-up visit, study staff identified important contacts. Contacts that participants communicated with at least twice by call or text in the past month were considered important. For each important contact, participants answered seven contextual questions about their type of relationship, whether they ever drank alcohol with this person, the drinking status of the contact, expectations about whether the contact would drink in their presence, recovery status of contact, level of supportiveness of contact, and affective experiences with the contact.

While enrolled, participants completed four brief daily ecological momentary assessments (7–10 questions). The first item assessed alcohol use (date and time of any unreported drinking episodes). The remaining EMA questions were used as features in other studies [10,11], but were outside the scope of this cellular communication sensing study. Additional sensing data streams and self-report measures were collected for the parent grant. We compensated participants up to $115 per month for completing study tasks (i.e., EMAs, monthly follow-up visits and sharing sensing data) and $66 per month to offset the cost of their cellphone plan. The full study protocol is available on our OSF page (https://osf.io/wgpz9/).

### Ethics

All procedures were approved by the University of Wisconsin-Madison Institutional Review Board (Study #2015-0780) and carried out in accordance with the principles of the Declaration of Helsinki. All participants provided written informed consent observed by a research assistant.

### Data analysis plan

#### Labels.

Our models predicted the probability of an alcohol lapse within a 24-hour window. Predictions were generated daily at 4 a.m., beginning on participants’ second study day and continuing for up to 3 months. Participants reported the date and hour of the start and end time of any alcohol use on the first item of the EMA. Participants demonstrated good adherence to our 4X daily EMA protocol (mean adherence = 78%) and provided at least one EMA on 95% of study days. Prediction windows were labeled as lapse if any alcohol use was reported in the 24-hour window. In total, there were 11,507 labeled prediction windows across all participants. Positive lapse labels were underrepresented (7.5%; 861/11,507).

#### Feature engineering.

Features were calculated using only data collected before the start of each prediction window to ensure our models were making true future predictions. We calculated a total of 181 features from four feature sets:

1. Cellular communication data. We filtered the raw communication data to include only communications with known context (i.e., people with whom they communicated with at least twice in a month and whom they provided self-report context about). Cellular communication features were engineered from all available data up to the start of each window. We used two feature scoring epochs (24 hours and 1 week before the start of the prediction window) to create a total of 142 features.
  Within each feature scoring epoch, we calculated two types of features: raw and difference features. Raw features represent the feature value calculated within a given feature scoring epoch. For example, the raw rate of incoming text messages in a 24-hour feature scoring epoch was calculated as the total number of incoming text messages in the 24 hours immediately preceding the start of the prediction window, divided by 24. Difference features capture participant-level changes from baseline. In other words, how does this feature value differ from what is typical for this person? Difference features were scored by subtracting the participant’s mean value (using all available data prior to the prediction window) from the associated raw feature value. For example, the difference feature for incoming text messages was calculated as the raw incoming text message rate minus the participant’s average incoming text message rate across all time on study.
2. Demographics. We created 2 numeric features for age and income, and 10 one-hot coded features for sex at birth (male, female), race and ethnicity (non-Hispanic White, Hispanic and/or not White), education (high school or less, some college, college degree), and marital status (married, not married, other).
3. Alcohol use characteristics. We created 21 features from baseline measures of alcohol use characteristics, including numeric features of DSM-5 AUD symptom count, craving, four abstinence self-efficacy subscales (negative affect, social, physical, and craving), number of individual alcohol counseling sessions, group alcohol counseling sessions, self-help meetings, and other mental health counseling sessions in the past 30 days, number of days in contact with supportive and unsupportive people in the past 30 days, and satisfaction with and confidence in their recovery over the past 30 days. We also created one-hot coded features for whether they have taken prescription medication for alcohol use disorder or mental health in the past 30 days and whether they had a goal of abstinence.
4. Psychiatric and personality characteristics. We created 6 numeric features from baseline measures of broad psychiatric and personality traits, including higher order personality dimensions of positive emotionality, negative emotionality, and constraint and psychiatric comorbidities of depression, anxiety, and stress.

Table 1 details the raw predictors, the feature set each predictor belongs to, feature engineering procedures used, scoring epochs (for cellular communication features only), and the total number of features derived from the raw predictor. We had no missing data across baseline measures of demographics, alcohol use characteristics, and psychiatric and personality characteristics. If no cellular communication data were observed during a feature scoring epoch that feature was scored 0, thus there were also no missing data among cellular communication features. Other feature engineering steps performed during cross-validation included standardizing all features and removing zero and near-zero variance features as determined from held-in data (code for our full data pre-processing pipeline is available on our OSF page [(https://osf.io/wgpz9/]).

**Table 1:** Raw predictors, feature set, response options, feature engineering methods, feature scoring epochs and total number of features for each feature set.

Raw Predictor	Feature Set	Response Options	Feature Engineering	Feature Scoring Epochs	Total Features
Originated	Cellular communication	Incoming, outgoing	Difference and raw rate counts for text messages and voice calls	24 hours and 1 week	16
Call duration	Cellular communication	Duration (in minutes)	Difference and raw rate sums of duration, difference and raw most recent duration	24 hours and 1 week	6
Call answered	Cellular communication	Yes, no	Difference and raw rate counts for unanswered incoming voice calls (i.e., response option = no)	24 hours and 1 week	4
Date/time of communication	Cellular communication	Date and time	Difference and raw rate counts for text messages and voice calls at night (10 pm-6am) and on weekends (Friday-Saturday)	24 hours^a^ and 1 week	12
Phone number	Cellular communication	Phone number	Difference and raw rate counts of unique phone numbers	24 hours and 1 week	4
Type of Relationship	Cellular communication	Family, friend, counselor or social worker, co-worker	Difference and raw rate counts of unique phone numbers	24 hours and 1 week	16
Have you drank alcohol with this person?	Cellular communication	Never/almost never, occasionally, almost always/always	Difference and raw rate counts of each response option	24 and 1 week	12
What is their drinking status?	Cellular communication	Drinker, non-drinker, don’t know	Difference and raw rate counts of each response option	24 and 1 week	12
Would you expect them to drink in your presence?	Cellular communication	Yes, no, uncertain	Difference and raw rate counts of each response option	24 and 1 week	12
Are they currently in recovery from drugs or alcohol?	Cellular communication	Yes, no, don’t know	Difference and raw rate counts of each response option	24 and 1 week	12
Are they supportive about your recovery goals?	Cellular communication	Supportive, unsupportive, mixed, neutral, don’t know	Difference and raw rate counts of each response option	24 and 1 week	20
How are your typical experiences with this person?	Cellular communication	Pleasant, unpleasant, mixed, neutral	Difference and raw rate counts of each response option	24 and 1 week	16
Age	Demographics	Numeric (years)			1
Sex at birth	Demographics	Male, female	One-hot coded		2
Race	Demographics	Non-Hispanic White, non-White and/or Hispanic	One-hot coded		2
Education	Demographics	High school or less, some college, college degree	One-hot coded		3
Income	Demographics	Numeric (dollars)			1
Marital Status	Demographics	Married, not married, other	One-hot coded		3
DSM-5 symptom count	Alcohol use	Numeric (4–11)			1
Craving	Alcohol use	Numeric (0–30)			1
Abstinence self-efficacy: Negative affect, social, physical, and craving subscales	Alcohol use	Numeric (0–20)			4
Number of individual alcohol counseling sessions attended (past 30 days)	Alcohol use	Numeric			1
Number of group alcohol counseling sessions attended (past 30 days)	Alcohol use	Numeric			1
Number of self-help group meetings attended (past 30 days)	Alcohol use	Numeric			1
Number of other mental health counseling sessions attended (past 30 days)	Alcohol use	Numeric			1
Number of days in contact with supportive people (past 30 days)	Alcohol use	Numeric			1
Number of days in contact with unsupportive people (past 30 days)	Alcohol use	Numeric			1
Taken prescribed medication for alcohol use disorder (past 30 days)	Alcohol use	Yes, no	One-hot coded		2
Taken prescribed medication for other mental health disorder (past 30 days)	Alcohol use	Yes, no	One-hot coded		2
Satisfaction with progress toward recovery goals (past 30 days)	Alcohol use	Numeric (0–4)			1
Confidence in abstinence ability (next 30 days)	Alcohol use	Numeric (0–4)			1
Has a goal of abstinence	Alcohol use	Yes, no, uncertain	One-hot coded		3
Personality dimensions (positive emotionality, negative emotionality, and constraint)	Personality/psychiatric	Numeric			3
Psychiatric conditions (depression, anxiety, and stress)	Personality/psychiatric	Numeric			3

Cellular communication features were scored over two feature scoring epochs (24 hours and 1 week before the start of the prediction window). Within each feature scoring epoch we calculated two types of features: raw and difference features. Raw features represent the raw feature value calculated within a scoring epoch (e.g., the rate count of text messages during the 24 hours immediately preceding the start of the prediction window). Difference features capture participant-level changes from their baseline scores (e.g., the participant’s average rate count of text messages across all time on study subtracted from the rate count in the preceding 24 hours).

^a^24-hour scoring epoch not used for calculating weekend features.

#### Model selection.

We included three candidate statistical algorithms in our model configurations: elastic net, random forest, and XGBoost. These algorithms differ in key ways related to flexibility, complexity, and their ability to accommodate non-linear and interactive relationships between features and lapse probability. Random forest and XGBoost algorithms can natively handle interactions, whereas elastic net assumes an additive data generating process. Thus, if true interactions exist between features, we would expect one of these tree-based methods to yield the best performance. However, if relationships are primarily additive, elastic net may perform better. Model configurations also differed on outcome resampling method (i.e., up-sampling and down-sampling of the outcome at ratios ranging from 5:1 to 1:1), hyperparameter values, and feature set.

We selected three final models. We selected the best performing full model that included all four feature sets (cellular communications, demographics, alcohol use characteristics, and psychiatric/personality characteristics). Because our feature set of interest was cellular communications we only selected from configurations that retained this feature set. We selected the best performing baseline model that included our three baseline feature sets (demographics, alcohol use characteristics, and psychiatric/personality characteristics) to assess the incremental predictive value of cellular communication beyond these baseline measures. Finally, we selected the best performing cellular communication model that included only the cellular communication feature set to evaluate the predictive signal captured in these features.

The best configuration for each of these models was selected using 6 repeats of 5-fold cross-validation. Participants were grouped so that all of their data were always in the held-in or held-out fold for a split, but never in both. Our performance metric was area under the receiver operating curve (auROC). Folds were stratified so that all folds contained comparable proportions of individuals who lapsed frequently (i.e., 10+ times).

#### Model evaluation.

We evaluated model performance for our three final models (full model, baseline model, cellular communication model) using a Bayesian hierarchical generalized linear model. Posterior distributions with 95% credible intervals (CI) were estimated from the 30 held-out folds using weakly informative, data-dependent priors to regularize and reduce overfitting: Residual SD ~ exponential(2.3); intercept (centered predictors) ~ normal(0.62, 1.1); fixed effects ~ normal(0, 2.27); covariance ~ decov(1, 1, 1, 1). Random intercepts were included for repeat and fold (nested within repeat). We reported the median posterior auROC and 95% CI for each model. We also specified a single model contrast: full model vs. baseline model. auROCs were logit-transformed and regressed on model contrast to estimate the probability that model performances differed systematically.

We refit our full model on the entire data set to identify the most important features. We quantified feature importance by examining the retained features (i.e., coefficient value > 0) in our final elastic net model and ordering them by absolute coefficient value. These values provide an estimate of the direction and magnitude of association between each predictor and the outcome, conditional on the other features retained.

## Results

### Participants

We enrolled 169 participants. Of these, 151 completed the first follow-up visit where the first cellular communication log download occurred. We excluded data from seven participants due to poor compliance providing communication data (i.e., deleting all text messages and voice calls prior to the download or not providing context information about important contacts). The final analytic sample included 144 participants. Table 2 provides the demographic characterization of our final sample. 56% of participants reported at least one lapse while on study.

**Table 2:** Demographic characterization.

	N	%	M	SD	Range
Age			40.4	11.8	21-72
Sex at Birth					
Female	74	51.4			
Male	70	48.6			
Race					
American Indian/Alaska Native	3	2.1			
Asian	2	1.4			
Black/African American	8	5.6			
White/Caucasian	125	86.8			
Other/Multiracial	6	4.2			
Hispanic, Latino, or Spanish origin					
Yes	3	2.1			
No	141	97.9			
Education					
Less than high school	1	0.7			
High school or GED	14	9.7			
Some college	39	27.1			
2-Year degree	13	9.0			
College degree	55	38.2			
Advanced degree	22	15.3			
Employment					
Employed full-time	70	48.6			
Employed part-time	25	17.4			
Full-time student	7	4.9			
Homemaker	1	0.7			
Disabled	7	4.9			
Retired	8	5.6			
Unemployed	15	10.4			
Temporarily laid off or on leave	3	2.1			
Other, not otherwise specified	8	5.6			
Personal Income			$35,050	$32,069	$0-200,000
Marital Status					
Never married	63	43.8			
Married	32	22.2			
Divorced	42	29.2			
Separated	5	3.5			
Widowed	2	1.4			
N = 144					

### Communications

Participants had an average of 26 important contacts (range 2–113) that were contextualized with self-report information. We obtained a total of 375,912 contextualized communications across participants. Participants had, on average, 2,610 contextualized communications (range = 109–14,225) averaging to about 33 communications per day (range 3–278).

### Model evaluation

**Fig 1:**
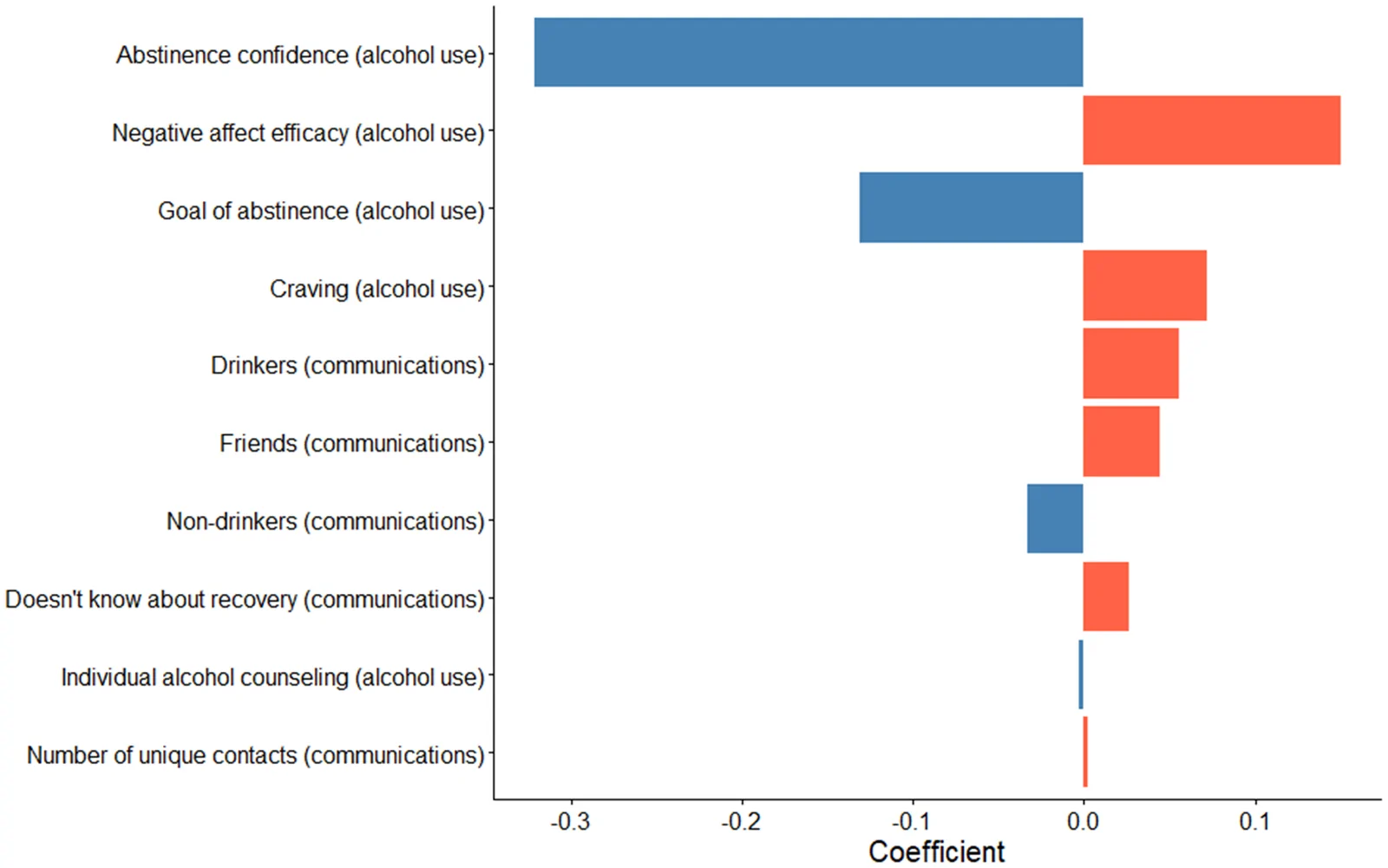
Global feature importance (elastic net coefficient) for the full model. Features are ordered by absolute coefficient value. Bars with negative coefficient values (in blue) represent features that, on average, lower lapse risk. Bars with positive coefficient values (in red) represent features that, on average, increase risk. Alcohol use features were collected via self-report measures at baseline. Communication features were engineered from contextualized cellular communications.

The median posterior auROC for the best performing full model was 0.674, 95% CI [0.639, 0.707]. The final model fit on the full dataset retained 10 features (Fig 1). The top four features were baseline alcohol use characteristics. Two of these (abstinence confidence and having a goal of abstinence) emerged as important protective factors. Abstinence self-efficacy when experiencing negative affect and craving emerged as important risk factors. Communication features were also retained in the final model. Frequency of communications with drinkers, friends, and people unaware of the individual’s recovery goals, and number of unique contacts emerged as important risk factors. Frequency of communications with non-drinkers, conversely, was an important protective factor. All communication features retained in the full model were scored over a 1-week feature scoring epoch.

We conducted a model comparison between the full model and a model that only considered baseline measures (alcohol use characteristics, demographics, and psychiatric and personality characteristics) to assess the incremental predictive value of cellular communication features beyond these baseline measures. The baseline model achieved slightly higher performance compared to the full model (median auROC = 0.687, 95% CI [0.653, 0.718]). However, the median difference in auROC was only 0.013 and the Bayesian model comparison showed weak evidence (73% probability) that the baseline model performed better than the full model.

Finally, our best performing model that included only cellular communication features achieved a median auROC of 0.588, 95% CI [0.550, 0.624]. 100% of the sampled posteriors were above 0.5 indicating strong evidence that the model is performing above chance performance (i.e., it is capturing some risk-relevant signal in the data). Four of the five cellular communication features retained in the full model were also retained in the cellular communication model. Number of unique contacts did not get retained in the cellular communication model, although it was retained in the full model with a very small coefficient. No new important features beyond those retained in the full model emerged in the cellular communication model.

## Discussion

Our full model achieved fair performance, with an auROC of 0.67, indicating that some predictive signal was present. However, it did not offer incremental value beyond a baseline model that included only baseline self-report measures. Consistent with this, the four most important predictors in our model were all self-report variables: abstinence confidence, abstinence goal, negative affect efficacy, and craving. Only baseline features related to alcohol use characteristics were retained in the full model, reinforcing that self-reported alcohol-related and recovery constructs are strong predictors of future lapse risk [4,5].

Cellular communication data on its own performed poorly for prediction (auROC = 0.67). However, it still performed above what we would expect from a model performing at chance, suggesting there may be some signal in these data. Additionally, several communication features were retained in the final model with moderately sized coefficients. These included communications with drinkers, non-drinkers, friends, and people unaware of the participant’s recovery goals. Number of unique contacts was also retained in the model, though with a much smaller coefficient. Given that cellular communication usage may vary widely across demographic characteristics, psychiatric states, personality profiles, and alcohol use severity, we were surprised to find that these features did not appear to substantially interact with the baseline self-report measures. The elastic net algorithm outperformed the tree-based models, suggesting the relationship between features are primarily additive as opposed to interactive.

The finding that communications with drinkers increased lapse risk and communications with non-drinkers decreased lapse risk is consistent with prior literature [19,20]. Individuals who surround themselves with people who drink (and who may have even drunk with the individual in the past) may be more likely to experience exposure to drinking-related cues and opportunities to drink. Conversely, communicating with non-drinkers may reflect a more supportive social environment that promotes recovery. Additionally, given that alcohol use is often a social activity, it is not unexpected that increased communication with friends increased lapse risk.

We were surprised to find that communications with people who did not know about an individual’s recovery goals was also a risk factor. We expected it was more likely that communication with people supportive of recovery goals would emerge as a protective factor or that communication with people unsupportive of those goals would emerge as a risk factor. One possible explanation is that a lack of disclosure about being in recovery reflects low levels of trust, making it difficult to confide in others about something so personal. It could also reflect perceived or anticipated stigma, thereby capturing the other person’s beliefs or attitudes about substance use. Lastly, it could signal reduced access to recovery support, as individuals cannot provide support for goals they are unaware of. Future research could explore this finding further to better understand the mechanisms underlying this association.

In contrast to the contextual features, raw counts of calls and text messages and call durations were not retained in the final model. This implies that the quantity of communication may be less informative than the quality and social significance. Future research may benefit from collecting richer contextual data about communication contacts to better understand the social dynamics contributing to lapse risk.

Even with highly contextualized communication data, however, prediction may be limited by data sparsity. Many participants had few daily communications, and some had extended periods with no recorded interactions at all. Our study design may have further contributed to this limitation. We collected only phone and SMS text communications through the native smartphone app. In recent years, many individuals use private messaging apps (e.g., WhatsApp, Signal) or social media platforms (e.g., Facebook Messenger, Instagram) as their primary communication method [41]. Therefore, our dataset likely missed a substantial portion of participants’ communications. Future studies could explore whether incorporating communication data from additional platforms yields stronger predictive signal. It may also be worthwhile to use scoring epochs longer than one week. Our final model retained only cellular communication feature scored over the one-week feature scoring epoch. This is likely due to these longer epochs containing more data than the shorter 24-hour scoring epochs. Longer scoring epochs (e.g., two weeks or one month) would contain even more data and may better capture slow evolving risk-features, such as a gradually shrinking social circle. Both of these benefits could potentially improve model performance.

However, even with improved data collection methods and longer scoring epochs, sparsity may remain a challenge. Some people may simply not communicate frequently with others and others use services (e.g., Snapchat) that automatically delete messages. We also cannot eliminate the possibility that our participants were purposefully deleting text messages and call logs that they did not want us to see. This may be especially relevant for text messages (e.g., where drinking plans were made). Although the focus of this study was on the metadata and context of cellular communications, text message content, nevertheless, was collected during the cellular communication log download as part of the larger aims of the parent project. This raises the possibility of missed opportunity for capturing risk-relevant signal due to non-random patterns of missing data (e.g., when data missingness systematically increases during periods of lapse).

Our conclusions are limited by sample composition and size. Our participants were predominantly non-Hispanic White and all were from the Madison, Wisconsin area. We had poor representation of older adults (65+ years) which may have been due to our requirement that participants owned and used a smartphone. Smartphone ownership is generally high. As of 2025, roughly 91% of Americans own a smartphone, with at least 85% ownership across across gender, race/ethnicity, socioeconomic status, and geographic location subgroups [42]. One exception to this pattern is with older adults. Ownership rates dip down to 78% among those age 65 years and older [42].

In addition to a mostly homogeneous sample composition, our sample size was relatively small (*N* = 144). Although the large number of observations (11,507 prediction windows across participants) was sufficient for training machine learning models with low bias and variance, model evaluation was conducted on a limited subset of new individuals. As a result, these estimates reflect expected performance on individuals similar in composition to those in our sample. Thus, findings may not generalize to more diverse populations or to individuals in different geographic contexts.

We determined the presence of AUD using a self-report measure of DSM-5 symptoms, as opposed to a structured clinical interview. This may have had some impact on our threshold for establishing AUD. It is possible that some participants over-endorsed experiencing symptoms (e.g., endorsing symptoms that occur infrequently and do not reflect a pattern) and have milder forms of AUD than our target sample. However, they still likely have AUD given that only two symptoms are needed for an AUD diagnosis and we required four for eligibility. Furthermore, all participants were required to be pursuing abstinence, suggesting they perceive a problem with alcohol use.

We cannot entirely dismiss the potential value of cellular communication data for risk prediction. For example, researchers have successfully incorporated communication data into models with other sensing data (e.g., accelerometer, geolocation, and device usage) to detect current [43] and predict future [44] heavy drinking episodes in non-treatment seeking young adult populations. It is possible that in certain populations cellular communications may hold more signal. Young adults may have more frequent communication reducing sparsity concerns. Additionally, non-treatment seeking populations may be less likely to censor their data (i.e., deleting communications) when the drinking behavior is not at odds with their goals and/or values. However, even in these instances, the unique contribution of cellular communications beyond other sensing methods is unclear. Some communication features, such as outgoing call duration and the number of outgoing calls emerged in the top 20 important features for detecting current drinking episodes. Conversely, when predicting future drinking episodes, no communication features appeared in the top 20. Other sensing methods, like geolocation and accelerometer data, appeared to be more robustly important for both detection and prediction.

Other practical challenges in collecting call and text message data further limit the feasibility of this sensing method. For example, we obtained participants’ cellular communication data by downloading backups of their communication logs in person during their monthly follow-up visits. It is possible to collect cellular communication data in real time using apps installed on Android devices. However, Apple heavily restricts apps in its app store from accessing call and text message data, making real-time sensing of communications challenging (if not impossible) for IOS users. We conclude that other forms of social interaction characterization (e.g., engineering time spent with supportive contacts from geolocation data) are more worthwhile to pursue in future research.
