# Supplementary material for: Evaluating cellular communication sensing for lapse risk prediction during early recovery from alcohol use disorder: A longitudinal observational study
Source: PLoS One. 2026 Aug 21;21(8):e0355396. doi: 10.1371/journal.pone.0355396 (PMC13496408; doi:10.1371/journal.pone.0355396)
Supplement: Supplementary file 1 [file pone.0355396.s002.pdf]

Below, we delineate the reviewers' and editor's questions, concerns, and requests regarding our original submission, and we detail the ways in which we have addressed each item. All comments have been copied verbatim to avoid misinterpretation.

Editor

1. Please ensure that your manuscript meets PLOS ONE's style requirements, including those for file naming.

We have reviewed the PLOS One's style requirements and updated our manuscript accordingly. We have also ensured that our file names meet the requirements.

2. Please note that PLOS One has specific guidelines on code sharing for submissions in which author-generated code underpins the findings in the manuscript. In these cases, we expect all author-generated code to be made available without restrictions upon publication of the work. Please review our guidelines at https://journals.plos.org/plosone/s/materials-and-software-sharing#loc-sharing-code and ensure that your code is shared in a way that follows best practice and facilitates reproducibility and reuse.

We have uploaded a copy of our code to our OSF page following the recommended guidelines for code sharing. This OSF page is also referenced in our manuscript.

3. PLOS requires an ORCID iD for the corresponding author in Editorial Manager on papers submitted after December 6th, 2016. Please ensure that you have an ORCID iD and that it is validated in Editorial Manager. To do this, go to 'Update my Information' (in the upper left-hand corner of the main menu), and click on the Fetch/Validate link next to the ORCID field. This will take you to the ORCID site and allow you to create a new iD or authenticate a pre-existing iD in Editorial Manager.

We have ensured the ORCID ID for the corresponding author is entered and validated.

4. Please include a copy of Table 1 which you refer to in your text on page 9

We ensured Table 1 was included in the manuscript.

5. If the reviewer comments include a recommendation to cite specific previously published works, please review and evaluate these publications to determine whether they are relevant and should be cited. There is no requirement to cite these works unless the editor has indicated otherwise.

N/A

Reviewer 1

1. Overall, the paper lacks clear hypotheses on how authors expected, for instance individual number of social contacts, or a preference for certain types of social contacts (with people who drink, know about abstinence goals etc.), or change in types of contacts to affect individual capability of keeping abstinence goals. Exploring communication patterns based on what is known about the role of social contacts or lack thereof in addiction and comorbid psychiatric disorders would better explain the rationale for the study.

The lack of explicit hypotheses is consistent with the primary focus of this paper on prediction and the additional explanatory analyses as exploratory. Our selection and feature engineering using cellular communication data was clearly derived from domain expertise capitalizing on the literature on social context and social networks. Furthermore, we have now added a brief review of that literature to the introduction. However, we did not add explicit hypotheses given the combined prediction and exploratory framework of the paper.

2. Continued, compulsive intake of alcohol is a feature of addiction. Drinking every day does not necessarily mean that the person does not have abstinence as a goal. It may just as well reflect severity of the condition. Therefore, excluding an individual based on an assumption (not defined in the exclusion criteria) is methodologically problematic. It could both affect results and limit generalizability.

We revisited our exclusion criteria and found that the one participant removed for continued drinking did not provide any cellular communication data at their first month follow-up visit. We could therefore not include them in our analyses. We have updated our exclusion criteria accordingly: We enrolled 169 participants. Of these, 151 completed the first follow-up visit where the first cellular communication log download occurred. We excluded data from seven participants due to poor compliance providing communication data (i.e., deleting all of their voice calls and text messages prior to the download or not providing context information about important contacts). The final analytic sample included 144 participants. No participants were excluded on a basis of drinking frequency or assumptions about abstinence goals.

3. Handling of missing data is insufficiently explained. In the addiction field data is seldom missing at random. To the contrary, missing data is often associated with relapse. Is is also more common in those with more severe addictive disorders. Author describe that missing data was imputed. Please specify how data was imputed. Describe which variables were imputed and what methods were used. Was there any association between missing variables and problem severity (ex symptom count of reported alcohol use).

We previously included an imputation step in our data pre-processing pipeline. However, we have since removed this as it was unnecessary. Our data set did not have any missing data on model features. If no cellular communication data were observed during a feature scoring epoch (i.e., the individual had no calls or text messages) that feature was scored 0. We also had no missing data across baseline measures included in our models. We now make this more clear in our methods. That being said, we appreciate the point this reviewer makes. It is still possible that participants were deleting cellular communications in a non-random manner. We acknowledge this now in the discussion as a potential limitation.

4. The size of the explored cohort is limited (<150 individuals). Use of cellular communication varies widely across sex, age, possibly on whether one is employed or not (i.e. busy during the day) or not etc. The social function of different types of cellular communication is also expected to vary widely. This is mentioned in the discussion, but authors are also encouraged to reflect on whether lack of a significant association could be due to type 2 error?

We now make more clearly in the methods and discussion how our study design can capture interactions between demographics (and other baseline measures) and cellular communications. Specifically, two of the three algorithms we considered (Random forest and XGBoost) can natively handle interactions. Thus, if there were strong interactions between sex, age, or employment status and cellular communication features, these models would have emerged as having the top performance. To the contrary, our best performing model used a linear elastic net algorithm. This suggests the relationship between features are primarily additive as opposed to interactive.

We also now mention our sample size as a potential limitation and discuss implications of this in our discussion.

5. Individual characteristics, including personality traits, potential comorbid psychiatric conditions/ symptoms such as anxiety or depression (if available) would be informative to add to the baseline and follow up measures and potentially inform changes in communication patterns.

We appreciate the suggestion and have added psychiatric and personality features to our model. Specifically, we now include three higher order dimensions of personality from the Multidimensional Personality Questionnaire Brief Form (positive emotionality, negative emotionality, and constraint) and three broad psychiatric conditions from the Depression Anxiety Stress Scale (Depression, Anxiety, and stress).

6. Isolating oneself, to a degree that is discernable in social behavior as part of increased anxiety and depression, craving may take longer than two weeks. Could authors explain how the different time windows performed and were interpreted? Could changes be associated with traits such as impulsivity, low mood, anxiety? Are data on these aspects available. Have interactions and potential mediation been explored?

We considered two different scoring epochs (24 hours and one week) to capture proximal and more distal patterns of communications precipitating lapse. It is possible that scoring features over longer epochs (e.g., two weeks or one month) could better capture slower-evolving processes that could be seen in communication data. However, because participants contributed, at most, three months of data, longer scoring epochs were not feasible. Specifically, a two week scoring epoch would mean that the first two weeks of observations (1/6th of participants data) would be predicted using less than two weeks of data. As a result these features would not be scored as intended or equivalent to future observations. We mention increasing the length of scoring epochs as a potential future direction in our discussion.

Features scored on the two time windows (24 hours and one week) could interact with communication and baseline features (including added psychiatric and personality measures) in two of our candidate statistical algorithms (XGBoost and random forest). If changes in traits such as depression and anxiety were associated with changes in communication features we would expect these models that natively handle interactions to outperform the linear elastic net model. However, our elastic net model, which assumes an additive data generating process, performed best. This suggests there were not notable interactions.

We now report in our results that the communication features retained in the final full model were all features scored over the 1-week feature scoring epoch. We also now comment on this and its implications in the discussion as well.

Given that only features scored over the one week scoring epoch were retained we did not perform any follow-up analyses on the different time windows.

7. Goal of abstinence, abstinence confidence tend to increase with abstinence time - the longer the abstinence the higher the continued goal of abstinence and the confidence. Was data on abstinence time available? Could these predictors be considered as time varying or maybe consider interaction terms?

Our baseline models included demographics, personality, and alcohol use characteristics at baseline and served as a model against which to test the incremental benefits of cellular communication beyond characteristics at study start. Given this, the only time-varying features in the model were features derived from the cellular communication features. Although we could measure time since last drink and include it as a feature this would not be consistent with the focus of the augmented model on time-varying communication features and evaluating the unique contribution of those communication features. Thus, we chose not to include abstinence duration as a time-varying feature in the model consistent with our decision to exclude many other time-varying features that could have been included (e.g., ecological momentary assessment and geolocation data) because they are outside the scope of this study.

8. Results reflect what is already known about craving and negative affect being positively associated with relapse risk. However, communication with someone who doesn't know about recovery is a novel finding and needs to be better explained. This association would be interesting to explore further.

We are unable to speak more about this feature, beyond its positive association with lapse risk (i.e., more frequent contacts with people who do not know the individual is in recovery increase lapse risk), in the context of our current analyses. We highlight this as an unexpected finding worth exploring more in future research in our discussion section.

9. Social isolation and lack of significant social support are important factors associated with recovery. Number of contacts varied widely (between 2 and >100!) in the population. I would expect increased social isolation (less communication, changes in communication pattern) in a the more social individuals (who have many contacts) to be a sign of increased depression or anxiety – which in turn could be associated with relapse. Could authors describe patterns of social contacts and change over time more clearly? Was number of contacts considered? Was change in communication, depending on number of contacts of any relevance?

While total number of contacts varied widely across participants, we did not include absolute number of contacts as a predictive feature. Doing so would have required using information not yet observed at the prediction time point (e.g., the total number of unique contacts communicated with over the full study period), and would introduce leakage from future data into the model.

We did consider relative (within-subject) changes in number of unique contacts and number of communications by engineering what we refer to in the manuscript as difference scores. Difference scores allow us

to create a feature that compares the number of contacts (or any other communication feature) during a given scoring epoch to that individual's typical number of contacts (across all study days up until the prediction time point). This allowed us to capture changes in communication relative to an individual's typical level of communication without using information from the future. We have updated the feature engineering section of the manuscript to more clearly describe these features.

Reviewer 2

1. Please include number of participants excluded after recruitment, the missing data rates, data preprocessing steps for the study.

We have included the requested information in the methods section. We also provide the excerpts from the manuscript here for easy reference.

We enrolled 169 participants. Of these, 151 completed the first follow-up visit where the first cellular communication log download occurred. We excluded data from seven participants due to poor compliance providing communication data (i.e., deleting all voice calls and text messages prior to the download or not providing context information about important contacts). The final analytic sample included 144 participants.

We had no missing data across baseline measures of demographics, alcohol use characteristics, and psychiatric and personality characteristics. If no cellular communication data were observed during a feature scoring epoch that feature was scored 0, thus there were also no missing data among cellular communication features. Other feature engineering steps performed during cross-validation included standardizing all features and removing zero and near-zero variance features as determined from held-in data (code for our full data pre-processing pipeline is available on our OSF page [(https://osf.io/wgpz9/])).

2. Some of the methodological concerns include - all the recruitment occurring from one Geographical location (Madison, WI) only, which limits generalizability, self reported DSM-5 symptoms for Alcohol use disorder rather than a structured clinical interview- patients don't have the clinical knowledge to detect symptom's accurately and reliably, Smartphone requirement introduces a selection bias.

We now comment on these limitations in our discussion section.

3. Ecological Momentary assessment compliance was not reported- which could introduce label misclassification. Alcohol use reporting may suffer from recall bias and social desirability bias.

We now include adherence rates to our EMA protocol. Specifically, participants demonstrated good adherence to our 4X daily EMA protocol (mean adherence = 78%) and provided at least one EMA on 95% of study days.

4. The method could have been strengthened by explaining how 192 participants is sufficient for power of study- in the setting of machine learning.

We appreciate this suggestion for strengthening our method justification. We now comment on this in the discussion.
