# Supplementary material for: Evaluating cellular communication sensing for lapse risk prediction during early recovery from alcohol use disorder: A longitudinal observational study
Source: PLoS One. 2026 Aug 21;21(8):e0355396. doi: 10.1371/journal.pone.0355396 (PMC13496408; doi:10.1371/journal.pone.0355396)
Supplement: Peer review history

# Peer review: Decision Letter 0

**DOI:** 10.1371/journal.pone.0355396.r001

**Authors:** Souparno Mitra

17 Mar 2026

Dear Dr. Curtin,

Thank you for submitting your manuscript to PLOS ONE. After careful consideration, we feel that it has merit but does not fully meet PLOS ONE’s publication criteria as it currently stands. Therefore, we invite you to submit a revised version of the manuscript that addresses the points raised during the review process.

==============================

**ACADEMIC EDITOR:** **Please address reviewers comments and resubmit for further consideration for publication.**

==============================

Please submit your revised manuscript by May 01 2026 11:59PM. If you will need more time than this to complete your revisions, please reply to this message or contact the journal office at plosone@plos.org. When you're ready to submit your revision, log on to https://www.editorialmanager.com/pone/ and select the 'Submissions Needing Revision' folder to locate your manuscript file.

- A letter that responds to each point raised by the academic editor and reviewer(s). You should upload this letter as a separate file labeled 'Response to Reviewers'.
- A marked-up copy of your manuscript that highlights changes made to the original version. You should upload this as a separate file labeled 'Revised Manuscript with Track Changes'.
- An unmarked version of your revised paper without tracked changes. You should upload this as a separate file labeled 'Manuscript'.

If you would like to make changes to your financial disclosure, please include your updated statement in your cover letter. Guidelines for resubmitting your figure files are available below the reviewer comments at the end of this letter.

If applicable, we recommend that you deposit your laboratory protocols in protocols.io to enhance the reproducibility of your results. Protocols.io assigns your protocol its own identifier (DOI) so that it can be cited independently in the future. For instructions see: https://journals.plos.org/plosone/s/submission-guidelines#loc-laboratory-protocols. Additionally, PLOS ONE offers an option for publishing peer-reviewed Lab Protocol articles, which describe protocols hosted on protocols.io. Read more information on sharing protocols at https://plos.org/protocols?utm_medium=editorial-email&utm_source=authorletters&utm_campaign=protocols.

We look forward to receiving your revised manuscript.

Kind regards,

Souparno Mitra, M.D.

Academic Editor

PLOS One

Journal Requirements:

When submitting your revision, we need you to address these additional requirements.

1. Please ensure that your manuscript meets PLOS ONE's style requirements, including those for file naming. The PLOS ONE style templates can be found at

https://journals.plos.org/plosone/s/file?id=wjVg/PLOSOne_formatting_sample_main_body.pdf and

https://journals.plos.org/plosone/s/file?id=ba62/PLOSOne_formatting_sample_title_authors_affiliations.pdf

2. Please note that PLOS One has specific guidelines on code sharing for submissions in which author-generated code underpins the findings in the manuscript. In these cases, we expect all author-generated code to be made available without restrictions upon publication of the work. Please review our guidelines at https://journals.plos.org/plosone/s/materials-and-software-sharing#loc-sharing-code and ensure that your code is shared in a way that follows best practice and facilitates reproducibility and reuse.

3. PLOS requires an ORCID iD for the corresponding author in Editorial Manager on papers submitted after December 6th, 2016. Please ensure that you have an ORCID iD and that it is validated in Editorial Manager. To do this, go to ‘Update my Information’ (in the upper left-hand corner of the main menu), and click on the Fetch/Validate link next to the ORCID field. This will take you to the ORCID site and allow you to create a new iD or authenticate a pre-existing iD in Editorial Manager.

4. Please include a copy of Table 1 which you refer to in your text on page 9

5. If the reviewer comments include a recommendation to cite specific previously published works, please review and evaluate these publications to determine whether they are relevant and should be cited. There is no requirement to cite these works unless the editor has indicated otherwise.

[Note: HTML markup is below. Please do not edit.]

Reviewers' comments:

Reviewer's Responses to Questions

**Comments to the Author**

1. Is the manuscript technically sound, and do the data support the conclusions?

Reviewer #1: Yes

Reviewer #2: Partly

**********

2. Has the statistical analysis been performed appropriately and rigorously?

Reviewer #1: Yes

Reviewer #2: I Don't Know

**********

3. Have the authors made all data underlying the findings in their manuscript fully available?

The PLOS Data policy

Reviewer #1: Yes

Reviewer #2: Yes

**********

4. Is the manuscript presented in an intelligible fashion and written in standard English?

Reviewer #1: Yes

Reviewer #2: Yes

**********

Reviewer #1: Thank you for the opportunity to review this article.

The use of information that is not dependent on self-report is an interesting and potentially useful approach.

Authors used cellular communication patterns as a measure of social functioning to predict relapse.

However, cellular communication, without a more qualitative, individualized and stratified approach, is not likely to be particularly informative. This is reflected in the results. Overall, the paper lacks clear hypotheses on how authors expected, for instance individual number of social contacts, or a preference for certain types of social contacts (with people who drink, know about abstinence goals etc.), or change in types of contacts to affect individual capability of keeping abstinence goals.

Exploring communication patterns based on what is known about the role of social contacts or lack thereof in addiction and comorbid psychiatric disorders would better explain the rationale for the study.

Methods

Continued, compulsive intake of alcohol is a feature of addiction. Drinking every day does not necessarily mean that the person does not have abstinence as a goal. It may just as well reflect severity of the condition. Therefore, excluding an individual based on an assumption (not defined in the exclusion criteria) is methodologically problematic. It could both affect results and limit generalizability.

Handling of missing data is insufficiently explained. In the addiction field data is seldom missing at random. To the contrary, missing data is often associated with relapse. Is is also more common in those with more severe addictive disorders. Author describe that missing data was imputed.

Please specify how data was imputed. Describe which variables were imputed and what methods were used. Was there any association between missing variables and problem severity (ex symptom count of reported alcohol use).

The size of the explored cohort is limited (<150 individuals). Use of cellular communication varies widely across sex, age, possibly on whether one is employed or not (i.e. busy during the day) or not etc. The social function of different types of cellular communication is also expected to vary widely.

This is mentioned in the discussion, but authors are also encouraged to reflect on whether lack of a significant association could be due to type 2 error?

Individual characteristics, including personality traits, potential comorbid psychiatric conditions/ symptoms such as anxiety or depression (if available) would be informative to add to the baseline and follow up measures and potentially inform changes in communication patterns.

Isolating oneself, to a degree that is discernable in social behavior as part of increased anxiety and depression, craving may take longer than two weeks.

Could authors explain how the different time windows performed and were interpreted? Could changes be associated with traits such as impulsivity, low mood, anxiety? Are data on these aspects available. Have interactions and potential mediation been explored?

Results

Goal of abstinence, abstinence confidence tend to increase with abstinence time - the longer the abstinence the higher the continued goal of abstinence and the confidence. Was data on abstinence time available? Could these predictors be considered as time varying or maybe consider interaction terms?

Results reflect what is already known about craving and negative affect being positively associated with relapse risk. However, communication with someone who doesn’t know about recovery is a novel finding and needs to be better explained.

This association would be interesting to explore further.

Social isolation and lack of significant social support are important factors associated with recovery. Number of contacts varied widely (between 2 and >100!) in the population. I would expect increased social isolation (less communication, changes in communication pattern) in a the more social individuals (who have many contacts) to be a sign of increased depression or anxiety – which in turn could be associated with relapse.

Could authors describe patterns of social contacts and change over time more clearly? Was number of contacts considered? Was change in communication, depending on number of contacts of any relevance?

Reviewer #2: - Please include number of participants excluded after recruitment, the missing data rates, data preprocessing steps for the study.

- Some of the methodological concerns include - all the recruitment occurring from one Geographical location (Madison, WI) only, which limits generalizability, self reported DSM-5 symptoms for Alcohol use disorder rather than a structured clinical interview- patients don't have the clinical knowledge to detect symptom's accurately and reliably, Smartphone requirement introduces a selection bias.

- Ecological Momentary assessment compliance was not reported- which could introduce label misclassification. Alcohol use reporting may suffer from recall bias and social desirability bias.

- The method could have been strengthened by explaining how 192 participants is sufficient for power of study- in the setting of machine learning.

**********

what does this mean?). If published, this will include your full peer review and any attached files.

If you choose “no”, your identity will remain anonymous but your review may still be made public.

**Do you want your identity to be public for this peer review?**  For information about this choice, including consent withdrawal, please see our Privacy Policy

Reviewer #1: No

Reviewer #2: **Yes:** Arun Prasad

**********

[NOTE: If reviewer comments were submitted as an attachment file, they will be attached to this email and accessible via the submission site. Please log into your account, locate the manuscript record, and check for the action link "View Attachments". If this link does not appear, there are no attachment files.]

To ensure your figures meet our technical requirements, please review our figure guidelines: https://journals.plos.org/plosone/s/figures

You may also use PLOS’s free figure tool, NAAS, to help you prepare publication quality figures: https://journals.plos.org/plosone/s/figures#loc-tools-for-figure-preparation.

NAAS will assess whether your figures meet our technical requirements by comparing each figure against our figure specifications.

# Author comment: Author response to Decision Letter 1

**DOI:** 10.1371/journal.pone.0355396.r002

1 May 2026

Below, we delineate the reviewers’ and editor’s questions, concerns, and requests regarding our original

submission, and we detail the ways in which we have addressed each item. All comments have been

copied verbatim to avoid misinterpretation. We also include this response as a pdf in the submitted materials to maintain its formatting in that pdf.

Editor

1. Please ensure that your manuscript meets PLOS ONE’s style requirements, including those for

file naming.

We have reviewed the PLOS One’s style requirements and updated our manuscript accordingly. We

have also ensured that our file names meet the requirements.

2. Please note that PLOS One has specific guidelines on code sharing for submissions in which

author-generated code underpins the findings in the manuscript. In these cases, we expect all

author-generated code to be made available without restrictions upon publication of the work.

Please review our guidelines at

https://journals.plos.org/plosone/s/materials-and-software-sharing#loc-sharing-code and ensure

that your code is shared in a way that follows best practice and facilitates reproducibility and reuse.

We have uploaded a copy of our code to our OSF page following the recommended guidelines for code

sharing. This OSF page is also referenced in our manuscript.

3. PLOS requires an ORCID iD for the corresponding author in Editorial Manager on papers

submitted after December 6th, 2016. Please ensure that you have an ORCID iD and that it is

validated in Editorial Manager. To do this, go to ‘Update my Information’ (in the upper left-hand

corner of the main menu), and click on the Fetch/Validate link next to the ORCID field. This will

take you to the ORCID site and allow you to create a new iD or authenticate a pre-existing iD in

Editorial Manager.

We have ensured the ORCID ID for the corresponding author is entered and validated.

4. Please include a copy of Table 1 which you refer to in your text on page 9

We ensured Table 1 was included in the manuscript.

5. If the reviewer comments include a recommendation to cite specific previously published works,

please review and evaluate these publications to determine whether they are relevant and should

be cited. There is no requirement to cite these works unless the editor has indicated otherwise.

N/A

Reviewer 1

1. Overall, the paper lacks clear hypotheses on how authors expected, for instance individual

number of social contacts, or a preference for certain types of social contacts (with people who

drink, know about abstinence goals etc.), or change in types of contacts to affect individual

capability of keeping abstinence goals. Exploring communication patterns based on what is known

about the role of social contacts or lack thereof in addiction and comorbid psychiatric disorders

would better explain the rationale for the study.

The lack of explicit hypotheses is consistent with the primary focus of this paper on prediction and

the additional explanatory analyses as exploratory. Our selection and feature engineering using cellular

communication data was clearly derived from domain expertise capitalizing on the literature on social

context and social networks. Furthermore, we have now added a brief review of that literature to the

introduction. However, we did not add explicit hypotheses given the combined prediction and exploratory

framework of the paper.

2. Continued, compulsive intake of alcohol is a feature of addiction. Drinking every day does not

necessarily mean that the person does not have abstinence as a goal. It may just as well reflect

severity of the condition. Therefore, excluding an individual based on an assumption (not defined

in the exclusion criteria) is methodologically problematic. It could both affect results and limit

generalizability.

We revisited our exclusion criteria and found that the one participant removed for continued drinking

did not provide any cellular communication data at their first month follow-up visit. We could therefore

not include them in our analyses. We have updated our exclusion criteria accordingly: We enrolled 169

participants. Of these, 151 completed the first follow-up visit where the first cellular communication

log download occurred. We excluded data from seven participants due to poor compliance providing

communication data (i.e., deleting all of their voice calls and text messages prior to the download or

not providing context information about important contacts). The final analytic sample included 144

participants. No participants were excluded on a basis of drinking frequency or assumptions about

abstinence goals.

3. Handling of missing data is insufficiently explained. In the addiction field data is seldom

missing at random. To the contrary, missing data is often associated with relapse. Is is also more

common in those with more severe addictive disorders. Author describe that missing data was

imputed. Please specify how data was imputed. Describe which variables were imputed and what

methods were used. Was there any association between missing variables and problem severity (ex

symptom count of reported alcohol use).

We previously included an imputation step in our data pre-processing pipeline. However, we have since

removed this as it was unnecessary. Our data set did not have any missing data on model features. If no

cellular communication data were observed during a feature scoring epoch (i.e., the individual had no

calls or text messages) that feature was scored 0. We also had no missing data across baseline measures

included in our models. We now make this more clear in our methods. That being said, we appreciate

the point this reviewer makes. It is still possible that participants were deleting cellular communications

in a non-random manner. We acknowledge this now in the discussion as a potential limitation.

4. The size of the explored cohort is limited (<150 individuals). Use of cellular communication

varies widely across sex, age, possibly on whether one is employed or not (i.e. busy during the

day) or not etc. The social function of different types of cellular communication is also expected

to vary widely. This is mentioned in the discussion, but authors are also encouraged to reflect on

whether lack of a significant association could be due to type 2 error?

We now make more clearly in the methods and discussion how our study design can capture interactions

between demographics (and other baseline measures) and cellular communications. Specifically, two

of the three algorithms we considered (Random forest and XGBoost) can natively handle interactions.

Thus, if there were strong interactions between sex, age, or employment status and cellular communication

features, these models would have emerged as having the top performance. To the contrary,

our best performing model used a linear elastic net algorithm. This suggests the relationship between

features are primarily additive as opposed to interactive.

We also now mention our sample size as a potential limitation and discuss implications of this in our

discussion.

5. Individual characteristics, including personality traits, potential comorbid psychiatric

conditions/ symptoms such as anxiety or depression (if available) would be informative to add to

the baseline and follow up measures and potentially inform changes in communication patterns.

We appreciate the suggestion and have added psychiatric and personality features to our model. Specifically,

we now include three higher order dimensions of personality from the Multidimensional Personality

Questionnaire Brief Form (positive emotionality, negative emotionality, and constraint) and three broad

psychiatric conditions from the Depression Anxiety Stress Scale (Depression, Anxiety, and stress).

6. Isolating oneself, to a degree that is discernable in social behavior as part of increased anxiety

and depression, craving may take longer than two weeks. Could authors explain how the different

time windows performed and were interpreted? Could changes be associated with traits such as

impulsivity, low mood, anxiety? Are data on these aspects available. Have interactions and

potential mediation been explored?

We considered two different scoring epochs (24 hours and one week) to capture proximal and more distal

patterns of communications precipitating lapse. It is possible that scoring features over longer epochs

(e.g., two weeks or one month) could better capture slower‑evolving processes that could be seen in

communication data. However, because participants contributed, at most, three months of data, longer

scoring epochs were not feasible. Specifically, a two week scoring epoch would mean that the first two

weeks of observations (1/6th of participants data) would be predicted using less than two weeks of data.

As a result these features would not be scored as intended or equivalent to future observations. We

mention increasing the length of scoring epochs as a potential future direction in our discussion.

Features scored on the two time windows (24 hours and one week) could interact with communication

and baseline features (including added psychiatric and personality measures) in two of our candidate

statistical algorithms (XGBoost and random forest). If changes in traits such as depression and anxiety

were associated with changes in communication features we would expect these models that natively

handle interactions to outperform the linear elastic net model. However, our elastic net model, which

assumes an additive data generating process, performed best. This suggests there were not notable

interactions.

We now report in our results that the communication features retained in the final full model were all

features scored over the 1-week feature scoring epoch. We also now comment on this and its implications

in the discussion as well.

Given that only features scored over the one week scoring epoch were retained we did not perform any

follow-up analyses on the different time windows.

7. Goal of abstinence, abstinence confidence tend to increase with abstinence time - the longer

the abstinence the higher the continued goal of abstinence and the confidence. Was data on

abstinence time available? Could these predictors be considered as time varying or maybe consider

interaction terms?

Our baseline models included demographics, personality, and alcohol use characteristics at baseline and

served as a model against which to test the incremental benefits of cellular communication beyond

characteristics at study start. Given this, the only time-varying features in the model were features

derived from the cellular communication features. Although we could measure time since last drink

and include it as a feature this would not be consistent with the focus of the augmented model on

time-varying communication features and evaluating the unique contribution of those communication

features. Thus, we chose not to include abstinence duration as a time-varying feature in the model

consistent with our decision to exclude many other time-varying features that could have been included

(e.g., ecological momentary assessment and geolocation data) because they are outside the scope of this

study.

8. Results reflect what is already known about craving and negative affect being positively

associated with relapse risk. However, communication with someone who doesn’t know about

recovery is a novel finding and needs to be better explained. This association would be interesting

to explore further.

We are unable to speak more about this feature, beyond its positive association with lapse risk (i.e.,

more frequent contacts with people who do not know the individual is in recovery increase lapse risk),

in the context of our current analyses. We highlight this as an unexpected finding worth exploring more

in future research in our discussion section.

9. Social isolation and lack of significant social support are important factors associated with

recovery. Number of contacts varied widely (between 2 and >100!) in the population. I would

expect increased social isolation (less communication, changes in communication pattern) in a the

more social individuals (who have many contacts) to be a sign of increased depression or anxiety –

which in turn could be associated with relapse. Could authors describe patterns of social contacts

and change over time more clearly? Was number of contacts considered? Was change in

communication, depending on number of contacts of any relevance?

While total number of contacts varied widely across participants, we did not include absolute number

of contacts as a predictive feature. Doing so would have required using information not yet observed

at the prediction time point (e.g., the total number of unique contacts communicated with over the full

study period), and would introduce leakage from future data into the model.

We did consider relative (within-subject) changes in number of unique contacts and number of communi-

cations by engineering what we refer to in the manuscript as difference scores. Difference scores allow us

to create a feature that compares the number of contacts (or any other communication feature) during

a given scoring epoch to that individual’s typical number of contacts (across all study days up until the

prediction time point). This allowed us to capture changes in communication relative to an individual’s

typical level of communication without using information from the future. We have updated the feature

engineering section of the manuscript to more clearly describe these features.

Reviewer 2

1. Please include number of participants excluded after recruitment, the missing data rates, data

preprocessing steps for the study.

We have included the requested information in the methods section. We also provide the excerpts from

the manuscript here for easy reference.

We enrolled 169 participants. Of these, 151 completed the first follow-up visit where the first cellular

communication log download occurred. We excluded data from seven participants due to poor compli-

ance providing communication data (i.e., deleting all voice calls and text messages prior to the download

or not providing context information about important contacts). The final analytic sample included 144

participants.

We had no missing data across baseline measures of demographics, alcohol use characteristics, and

psychiatric and personality characteristics. If no cellular communication data were observed during a

feature scoring epoch that feature was scored 0, thus there were also no missing data among cellular

communication features. Other feature engineering steps performed during cross-validation included

5standardizing all features and removing zero and near-zero variance features as determined from held-

in data (code for our full data pre-processing pipeline is available on our OSF page [(https://osf.io/

wgpz9/]).

2. Some of the methodological concerns include - all the recruitment occurring from one

Geographical location (Madison, WI) only, which limits generalizability, self reported DSM-5

symptoms for Alcohol use disorder rather than a structured clinical interview- patients don’t have

the clinical knowledge to detect symptom’s accurately and reliably, Smartphone requirement

introduces a selection bias.

We now comment on these limitations in our discussion section.

3. Ecological Momentary assessment compliance was not reported- which could introduce label

misclassification. Alcohol use reporting may suffer from recall bias and social desirability bias.

We now include adherence rates to our EMA protocol. Specifically, participants demonstrated good

adherence to our 4X daily EMA protocol (mean adherence = 78%) and provided at least one EMA on

95% of study days.

4. The method could have been strengthened by explaining how 192 participants is sufficient for

power of study- in the setting of machine learning.

We appreciate this suggestion for strengthening our method justification. We now comment on this in

the discussion.

Attachment Submitted filename: Response to Reviewers.pdf

# Peer review: Decision Letter 1

**DOI:** 10.1371/journal.pone.0355396.r003

**Authors:** Souparno Mitra

21 Jul 2026

Evaluating Cellular Communication Sensing for Lapse Risk Prediction During Early Recovery from Alcohol Use Disorder: A Longitudinal Observational Study

PONE-D-26-04086R1

Dear Dr. Curtin,

We’re pleased to inform you that your manuscript has been judged scientifically suitable for publication and will be formally accepted for publication once it meets all outstanding technical requirements.

Within one week, you’ll receive an e-mail detailing the required amendments. When these have been addressed, you’ll receive a formal acceptance letter and your manuscript will be scheduled for publication.

An invoice will be generated when your article is formally accepted. Please note, if your institution has a publishing partnership with PLOS and your article meets the relevant criteria, all or part of your publication costs will be covered. Please make sure your user information is up-to-date by logging into Editorial Manager at Editorial Manager® and clicking the ‘Update My Information' link at the top of the page. For questions related to billing, please contact billing support.

If your institution or institutions have a press office, please notify them about your upcoming paper to help maximize its impact. If they’ll be preparing press materials, please inform our press team as soon as possible -- no later than 48 hours after receiving the formal acceptance. Your manuscript will remain under strict press embargo until 2 pm Eastern Time on the date of publication. For more information, please contact onepress@plos.org.

Kind regards,

Souparno Mitra, M.D.

Academic Editor

PLOS One

Additional Editor Comments (optional):

Reviewers' comments:

Reviewer's Responses to Questions

**Comments to the Author**

Reviewer #1: All comments have been addressed

Reviewer #2: All comments have been addressed

**********

2. Is the manuscript technically sound, and do the data support the conclusions?

Reviewer #1: Yes

Reviewer #2: Partly

**********

3. Has the statistical analysis been performed appropriately and rigorously?

Reviewer #1: I Don't Know

Reviewer #2: I Don't Know

**********

4. Have the authors made all data underlying the findings in their manuscript fully available?

The PLOS Data policy

Reviewer #1: Yes

Reviewer #2: Yes

**********

5. Is the manuscript presented in an intelligible fashion and written in standard English?

Reviewer #1: Yes

Reviewer #2: Yes

**********

Reviewer #1: Thank you for addressing my comments.

The paper has very much improved in this new revised version.

Please note a typo on page 26, line 435. I think you mean to write censor not sensor.

Reviewer #2: Thank you for addressing the concerns we had in the comments on the study "Evaluating Cellular Communication Sensing for Lapse Risk Prediction During Early Recovery from Alcohol Use Disorder: A Longitudinal Observational Study"

**********

what does this mean?). If published, this will include your full peer review and any attached files.

If you choose “no”, your identity will remain anonymous but your review may still be made public.

**Do you want your identity to be public for this peer review?**  For information about this choice, including consent withdrawal, please see our Privacy Policy

Reviewer #1: No

Reviewer #2: **Yes:** Arun Prasad

**********

# Editor report: Acceptance letter

**DOI:** 10.1371/journal.pone.0355396.r004

**Authors:** Souparno Mitra

PONE-D-26-04086R1

PLOS One

Dear Dr. Curtin,

I'm pleased to inform you that your manuscript has been deemed suitable for publication in PLOS One. Congratulations! Your manuscript is now being handed over to our production team.

At this stage, our production department will prepare your paper for publication. This includes ensuring the following:

* All references, tables, and figures are properly cited

* All relevant supporting information is included in the manuscript submission,

* There are no issues that prevent the paper from being properly typeset

You will receive further instructions from the production team, including instructions on how to review your proof when it is ready. Please keep in mind that we are working through a large volume of accepted articles, so please give us a few days to review your paper and let you know the next and final steps.

Lastly, if your institution or institutions have a press office, please let them know about your upcoming paper now to help maximize its impact. If they'll be preparing press materials, please inform our press team within the next 48 hours. Your manuscript will remain under strict press embargo until 2 pm Eastern Time on the date of publication. For more information, please contact onepress@plos.org.

You will receive an invoice from PLOS for your publication fee after your manuscript has reached the completed accept phase. If you receive an email requesting payment before acceptance or for any other service, this may be a phishing scheme. Learn how to identify phishing emails and protect your accounts at https://explore.plos.org/phishing.

If we can help with anything else, please email us at customercare@plos.org.

Thank you for submitting your work to PLOS ONE and supporting open access.

Kind regards,

PLOS ONE Editorial Office Staff

on behalf of

Dr. Souparno Mitra

Academic Editor

PLOS One
